# Supplementary material for: Non-Targeted Metabolomics Reveals Sorghum Rhizosphere-Associated Exudates are Influenced by the Belowground Interaction of Substrate and Sorghum Genotype
Source: Int J Mol Sci. 2019 Jan 19;20(2):431. doi: 10.3390/ijms20020431 (PMC6358735; doi:10.3390/ijms20020431)
Supplement: Supplementary file 1 [file ijms-20-00431-s001.zip › Figure S1 VolcanoPlotsMetabolitesofInterest.docx]

**Figure S1.** Volcano plots reflect metabolites of interest. Each plot was created within a substrate type using –log 10 of FDR adjusted p-values (y axis) and log_2_ fold changes (x axis) between each plant treatment (BTx623 and SC56) and no-plant control. Blue dots represent BTx623 whereas red dots represent SC56 in **a)** sand, **b)** clay and **c)** soil. Metabolites that are present at a value greater than 1.3 on the y axis (dashed gray line) and greater than 1 on the x axis (dashed gray line) are considered rhizosphere-associated exudates (RAEs) whereas metabolites greater than 1.3 on the y axis and less than -1 on the x axis (dashed gray line) are considered rhizosphere-abated metabolites (RAMs).

REMs

REMs

REMs

**a**

**b**

**c**
