# Supplementary material for: Non-Targeted Metabolomics Reveals Sorghum Rhizosphere-Associated Exudates are Influenced by the Belowground Interaction of Substrate and Sorghum Genotype
Source: Int J Mol Sci. 2019 Jan 19;20(2):431. doi: 10.3390/ijms20020431 (PMC6358735; doi:10.3390/ijms20020431)
Supplement: Supplementary file 1 [file ijms-20-00431-s001.zip › Table S1 SoilCharactaristicsofBulkSubstrates.docx]

**Table S1.** Soil characteristics of bulk substrates. Characteristics were analyzed using no-plant controls at 21 days after sowing and included the addition of Hoagland solution as a fertilizer.

| **Substrate** | **Sand** | **Clay** | **Soil** |
| --- | --- | --- | --- |
| Bulk Density (g/cm^3^) | 1.55 | 0.66 | 0.33 |
| Organic Matter (LOI^a^ %) | 0.2 | <0.1 | 50.1 |
| Cation Exchange Capacity (me/100g) | 2.6 | 8.0 | 16.8 |
| Gravimetric Soil Moisture (%) | 25.9 | 92.7 | 135.7 |
| Organic Carbon (%) | 0.019 | 0.067 | 24.537 |
| pH | 8.4 | 5.2 | 5.8 |
| Nitrogen (ppm) | 19 | 98 | 6178 |
| Phosphorous (ppm) | 12 | 50 | 107 |
| Potassium (ppm) | 44 | 604 | 315 |
| Sulfate (ppm) | 7 | 55 | 136 |
| Zinc (ppm) | 0.14 | 0.35 | 2.72 |
| Iron (ppm) | 3.9 | 32.4 | 25.2 |
| Manganese (ppm) | 0.5 | 9.3 | 4.2 |
| Copper (ppm) | 0.18 | 0.05 | 0.35 |
| Calcium (ppm) | 404 | 491 | 998 |
| Magnesium (ppm) | 62 | 175 | 346 |
| Sodium (ppm) | 12 | 27 | 35 |

^a^ loss on ignition
